# Intelligence

**Published:** 1828-09

**Authors:** 


					285
INTELLIGENCE.
MONTHLY REPORT OF PREVALENT DISEASES.
We have recently met with a good deal of fever, and in a considerable num-
ber of instances the disease has assumed an intermittent type. Notwith-
standing the wetness of the season, however, it does not appear to have been
npon the whole unhealthy. There has been much less than usual of diarrhoea,
and scarcely any cases coming under the description of cholera have fallen
.under our notice: indeed, it is only during the last ten days, since the weather
became warmer, that any form of bowel complaint has been common.
College of Surgeons.?Mr. Lawrence has been elected a member of the
Council of the College of Surgeons.
Examinations for the Degree of M.B. at Cambridge.
To the Editors of the London Medical and Physical Journal.
Gentlemen,?In your last Number, a correspondent, who signs himself
Verax, has given a list of questions which were submitted to the candidates
for the M.B. degree in the university of Cambridge in June last. He leaves
the public " to judge whether such an examination is inferior to any in
Europe in difficulty, and whether persons answering them fully on paper are not
qualified for their admission to their first degree." Now Verax is either un-
candid, or he is ignorant of the subject upon which he ventures to address you.
For it is notorious that many of the most difficult questions in these examina-
tions are generally not answered at all4, and they are selected in order that a
candidate of very superior qualifications may have an opportunity of distin-
guishing himself from the crowd, or that, by their subsequent publication, the
importance of an university education may be inferred from the difficult exami-
nation which the candidates are hastily presumed to have passed. But allow
me to state that, if only a few of these questions are answered, the candidate
still receives his degree. A printed list of all the questions is given to him
upon his admission, and, by subsequent reference or study, he may become
capable of understanding the whole. He then shews his examination with
confidence, and comments upon its severity; and his ability is estimated by a
test to which he has not in fact been fairly submitted.
I am, gentlemen, your obedient servant,
JUSTUS.
Remarkable Appearance in the Eyes of a Child.?One of the leading wonders
of the day in the French capital, to which the " seekers of the extraordinary"
have been lately attracted, has been an infant of three years of age, who was
said to have the words Napoleon Emperf.ur very clearly marked in her
eyes. Unwilling to allow this lusus to escape us, we took advantage of an
opportunity, afforded us by the kindness of Mr. Guthrie,* of judging for
ourselves. We confess we were before a little sceptical upon the subject.
The fact is simply this : The child has light blue eyes, the irides being very
strongly striated with irregular white lines, which have been thought to con-
stitute the above ominous words. In our opinion, it would require a very
* At this time she was not publicly exhibited.?E.
286 INTELLIGENCE.
poetical vision, and a great deal of imagination, to discover them. Some of
the lines certainly resemble letters, but we endeavoured in vain to make out
any distinct words. It is true we had no magnifying glass at hand, which the
mother assured us was necessary to make the letters clearly perceptible.
The French police have taken alarm, and have deemed it prudent to de-
prive the friends of various certificates which they had obtained from different
persons, asserting that they could with facility decipher the much dreaded
name. We remember that, some years ago, the name of Napoleon was said
to have been detected upon a hen's egg, in good round German text. The
times were then propitious for the occurrence of such a phenomenon ; but, if
it were to happen now, the ill-fated animal would probably be placed under
the strict surveillance of the guardians of public tranquillity. If the memory
of Bonaparte is not kept alive by any more alarming circumstance than the
appearance in the eyes of this very engaging infant, the Bourbon dynasty will
not be endangered.?We believe it is intended to make a public exhibition of
the child.
We are informed that Dr. Munro, of Edinburgh, frequently exhibited to
his class, about two years ago, a child, in whose eyes many persons imagined
they could trace the name and age of its father; and there is now living at
Hull a boy, who is reported to have his name, John While, very clearly marked
in the iris of each eye. In most persons who have light-coloured eyes, there
may be seen around the circle of the iris irregular lines and marks of various
forms ; and, in the above cases, these natural appearances have been tortured
into different words by the creative fancy of different individuals, either from
a love of the marvellous or from interested motives.
Spontaneous Dislocation of the Hip Joint.?A healthy boy, about fourteen
years of age, very imprudently amused himself by frequently remaining in the
water, bathing, for several hours a day. He became subject to rheumatic
affections of his joints, and complained of uneasiness in his hip, which pre-
vented him from moving without great pain. By care and appropriate treat-
ment, he soon recovered the use of the limb. He was now deemed conva-
lescent; but, after having passed a good night, he rose in the morning
perfectly lame, and his hip-joint was found to be dislocated. He had met
with no accident, nor was he conscious of having been at all restless during
the night. For what reason we know not, but no attempts were made to
reduce the dislocation, until a-year had elapsed. Every effort was then ex-
cited in vain to restore the parts to their natural situation, and the patient
now remains a complete cripple. The head of the thigh-bone may be plainly
felt on the dorsum of the ilium.
We presume that in this case dislocation occurred either from a relaxation
of the ligaments, or perhaps from a loss of muscular power, and brought on
by the imprudent habit which we have mentioned. Sir Astley Cooper
mentions similar cases in his splendid work on Dislocations.
Mineral Teeili.?There are many disadvantages attending the common mode
of preparing mineral teeth in one solid piece. M. Boullancourt, who
was long under the tuition of the famous Delabarre of Paris, has suc-
ceeded in mounting them exactlyin the same manner as the natural teeth.
By this means the disagreeable sensation and noise in the mouth, which exist'
Literary Notices, 8?c. 287
when they are formed of one solid piece, are obviated. They are light and
comfortable in the mouth, and of a very natural appearance.
M. Boullancourt has also made several very ingenious improvements in the
construction of artificial noses, and in different instruments for defective
palates.
Second Editions of Works.
To the Editors of the London Medical and Physical Journal.
YotJ will oblige a member of the College of Surgeons by giving insertion to
the following note.
Some time ago yow did me the honour to notice with approbation some
sentiments contained in a note, on the advantages we should derive by
authors publishing alterations and improvements in their second editions, in
the form of appendices. Since that period I am happy in being able to state,
Dr. Aiinott has set an example which deserves to be noticed. He has pub-
lished a second edition of his interesting work on Physics, and has had the
generosity to publish separately, in the form of Appendix, the alterations and
improvements contained therein. Were such a practice to prevail, the bene-
fit to the profession and public would be incalculable.
Literary Notices.
Observations on Fever. By R. Wade, Member of the Royal College of
Surgeons. Second edition. 1828.
We are glad to see that this little work, to which we formerly drew the
attention of our readers, has reached a second edition. It is expressly de-
signed for the use of students, and well calculated for its object. The author
has lived much among patients and pupils, and endeavours to carry his readers
to the bedside, describing diseases as they are to be found in actual practice,
and pointing out the appropriate remedies in a clear and perspicuous manner.
It would be foreign to our object to quote from a work avowedly elementary,
or to enter at large into details which do not profess to contain any thing-new;
but we recommend the work to young practitioners as containing various re-
marks calculated to prove useful to them.
Natural Theology; or, Evidences of the Existence and Attributes of The
Deity, collected from the Appearances of Nature. By William Paley,
d.d. Illustrated by a series of Plates and Explanatory Notes. By James
Paxton, Surgeon, Oxford. Second Edition.?t vols. 8vo. Vincent, Oxford,
1828.
It may appear to be travelling out of oar record to take any notice of such a
work as the present. But, as the author has drawn his numerous illustrations
and proofs from the marvellous machinery of auimated nature, the medical
student and practitioner will both derive instruction and amusement in the
perusal of it. Dr. Paley's work is too firmly established in public opinion to
require any formal praise at our hands. Mr. Paxton, the editor of the present
edition, deserves, however, our especial thanks for having considerably in-
creased the utility of the original work, by his graphic illustrations and expla-
natory and emendatory notes; for the want of which there was before snme
difficulty in fully comprehending the text, notwithstanding the simple perspi-
cuity of style which characterises all the writings of Dr. Paley,
METEOROLOGICAL JOURNAL,
From July 20th, to August 20\ih, 1828.
By Messrs. Harris and Co. Mathematical Instrument Makers, 50, High Holborn.
20
21
22
23
24
25
26
27
28
29
30
31
Auif.
T
2
3
4
6
6
7
8
9
10
11
12
13
14
15
16
17
18
o
Winds.
NW
sw
w
NW
NW
VVNW
WNW
W
SW
W
W
WNW
W
E
NNE
NW
W
NW
66 : 69 57 30.00 30.01 50 50 NW
NNE
S
NW
W
W
W
WNW
NW
N
ESE
NNW
NW
SW
W
W
W
w
NW
NW
W
W
W
WSW
W
SSE
KSE
NW
W
NW
NW
NW
Atmospheric Variations
2 p.m. 10 p.m.
Kaia
;Fine
Cloudy
Fine
Kain
Fine
Cloudy
Fine
Fair
Cloudy
Fine
Fine
Cloudy
Ruin
Fine
Cloud/
Fair
Kain
Fine
Hain
Fine
liain
Fair
Kain
Kain
Fine
Cloudy
Kain
Kain
Fair
Kain
Pine
Fair
Show'ry
Kain
Fine
Kain
Fine
Kain
Fair
Fiue
Kain
Fine
Fine
Kain
Fine
Fair
Kain
Fine
Kain
Fine
Kain
Fiue
The quantity of Rain fallen in the month of July, was 5 inches and 36.100ths.

				

